# Detection of opsonizing antibodies directed against a recently circulating *Bordetella pertussis* strain in paired plasma samples from symptomatic and recovered pertussis patients

**DOI:** 10.1038/s41598-018-30558-8

**Published:** 2018-08-13

**Authors:** Elise S. Hovingh, Betsy Kuipers, Axel A Bonačić Marinović, Hendrik Jan Hamstra, Danielle Hijdra, Lapo Mughini Gras, Inonge van Twillert, Ilse Jongerius, Cecile A. C. M. van Els, Elena Pinelli

**Affiliations:** 1 Centre for Infectious Disease Control, National Institute for Public Health and the Environment (RIVM), Bilthoven, The Netherlands; 2 Department of Medical Microbiology, University Medical Centre Utrecht, Utrecht, The Netherlands; 3 Department of Infectious Diseases and Immunology, Utrecht University, Utrecht, The Netherlands; 4 Present Address: Department of Immunopathology, Sanquin Research and Landsteiner Laboratory of the Academic Medical Centre, University of Amsterdam, Amsterdam, The Netherlands

## Abstract

Correlates of protection (CoPs) against the highly contagious respiratory disease whooping cough, caused by *Bordetella pertussis*, remain elusive. Characterizing the antibody response to this pathogen is essential towards identifying potential CoPs. Here, we evaluate levels, avidity and functionality of *B*. *pertussis-*specific-antibodies from paired plasma samples derived from symptomatic and recovered pertussis patients, as well as controls. Natural infection is expected to induce protective immunity. IgG levels and avidity to nine *B*. *pertussis* antigens were determined using a novel multiplex panel. Furthermore, opsonophagocytosis of a *B*. *pertussis* clinical isolate by neutrophils was measured. Findings indicate that following infection, *B*. *pertussis-*specific antibody levels of (ex-) pertussis patients waned, while the avidity of antibodies directed against the majority of studied antigens increased. Opsonophagocytosis indices decreased upon recovery, but remained higher than controls. Random forest analysis of all the data revealed that 28% of the opsonophagocytosis index variances could be explained by filamentous hemagglutinin- followed by pertussis toxin-specific antibodies. We propose to further explore which other *B*. *pertussis-*specific antibodies can better predict opsonophagocytosis. Moreover, other *B*. *pertussis-*specific antibody functions as well as the possible integration of these functions in combination with other immune cell properties should be evaluated towards the identification of CoPs against pertussis.

## Introduction

Whooping cough, a highly contagious respiratory disease caused by the Gram-negative bacterium *Bordetella pertussis*, has resurged despite high vaccine coverage^1,2^. In the 1990s, the whole cell pertussis vaccine was replaced by the current acellular pertussis vaccine (ACV) in many industrialized countries. The ACV effectively prevents disease. However, protective immunity is lost 4–7 years post-vaccination and this vaccine does not protect against transmission^3–5^ urging the development of an improved vaccine. Evaluating pertussis vaccine-induced protection poses a problem as correlates of protection (CoPs)^6^ against pertussis have not been defined. A CoP can be defined as an (immune) marker that statistically correlates with vaccine efficacy but is not necessarily mechanistically responsible for protection^6^. While high levels of anti-pertussis toxin (Ptx) have been shown to be indicative for protection against disease, no reliable threshold has been established^7–9^. It is widely accepted that a well-defined CoP will not be unveiled merely by monitoring the antibody levels induced by the ACV^10,11^. In addition to the quantity, the quality and functionality of antibodies should also be evaluated^12^. This has not been extensively studied in pertussis, as typically IgG levels and isotypes are measured^13^.

For pertussis, a CoP would ideally correlate with the inability of the bacterium to colonise the airways and would hence correlate with protection from transmission of this pathogen. One of the functions of antibodies is to facilitate the uptake of bacteria via Fc-γ-receptor-mediated opsonophagocytosis by, for example, neutrophils which play an important role in clearing *B*. *pertussis* during infection and thus in preventing colonisation^14^. The relevance of avidity, indicating the strength by which the antibody binds the antigen, in relation to opsonophagocytosis has not been documented for *B*. *pertussis*. However, antibody avidity has been shown to be important for opsonophagocytosis of other bacteria including *Streptococcus pneumoniae*^15^.

Natural infection with *B*. *pertussis* induces a strong immune response across all age groups^13^. Compared to vaccine-induced protection, it provides the longest protection from disease with estimates of up to 20 years^3^. In an attempt to identify CoPs, we evaluated the levels and quality of antibodies directed against nine different virulence factors as well as the functionality of *B*. *pertussis*-specific antibodies in paired plasma samples of symptomatic as well as recovered pertussis patients, who are expected to have protective immunity conferred by natural infection. These antigens included the well-studied ACV components, Ptx, pertactin (Prn), filamentous hemagglutinin (FHA) and fimbriae (Fim) 2 and 3 and the virulence factors lipooligosaccharide (LOS), Bordetella resistance to killing A (BrkA), virulence associated gene 8 (Vag8) as well as whole outer membrane vesicles (OMVs). The antibody functionality was assessed by means of an antibody-mediated opsonophagocytosis assay (OPA) using a recently circulating *B*. *pertussis* strain. Moreover, integrated analysis of the generated data on levels and avidity was performed in an attempt to identify *B*. *pertussis*-specific antibodies predictive for opsonophagocytosis of this pathogen as potential CoPs against pertussis.

## Results

### Waning of antigen-specific antibodies years after *B. pertussis* infection

**Figure 1:**
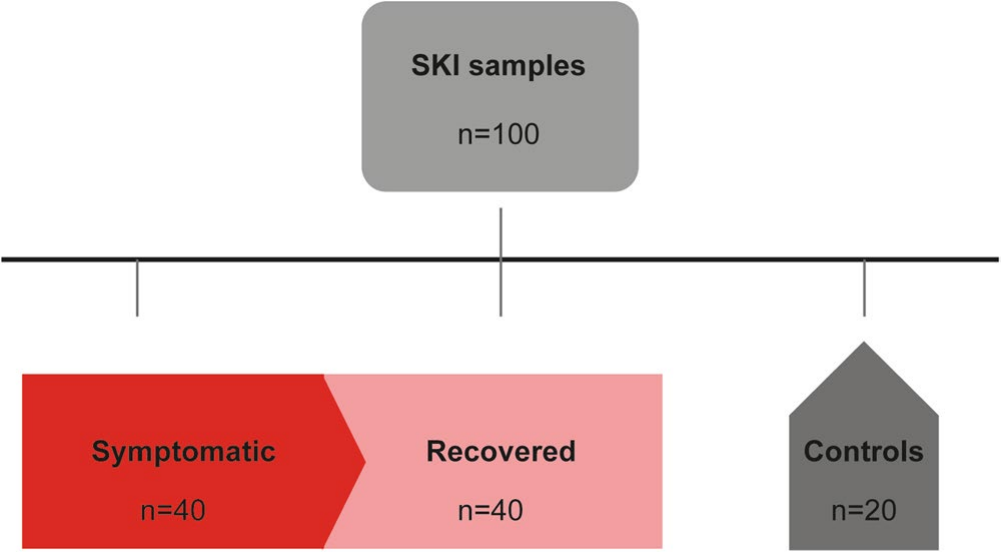
Schematic representation of SKI plasma samples used in this study. 100 plasma samples were selected from the SKI study which included 40 paired samples from symptomatic or recovered (ex) pertussis patients and 20 samples from controls.

To characterize the natural antibody response to *B*. *pertussis* we determined levels of IgG, not only against the well-studied vaccine antigens Ptx, FHA, Prn, Fim2 and Fim3^13,16^, but also against Vag8, BrkA, LOS and OMVs using paired plasma samples of symptomatic and recovered pertussis patients and controls (Fig. 1).

**Figure 2:**
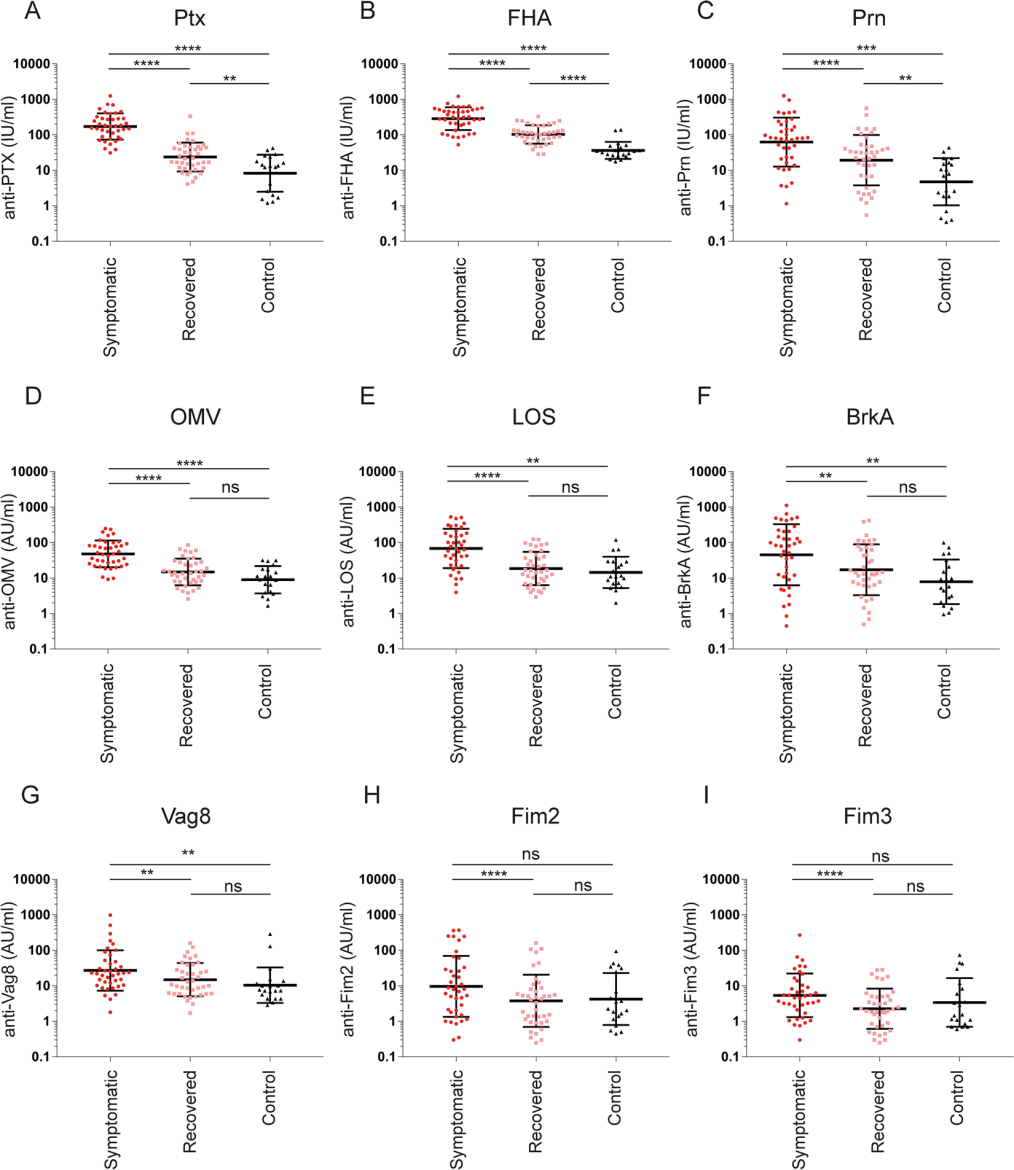
Levels of IgG antibodies directed against nine different pertussis antigens. Antibodies directed against (**A**) Ptx, (**B**) FHA, (**C**) Prn, (**D**) OMV, (**E**) LOS, (**F**) BrkA, (**G**) Vag8, (**H**) Fim3 and (**I**) Fim2 were determined using a 9-valent multiplex immunoassay platform using plasma samples collected from symptomatic (circles) and recovered (squares) pertussis patients as well as from controls (triangles). Statistical testing: one-way analysis of variance (ANOVA) followed by post-hoc tests (Welch Two Sample t-test or paired t-test). The false discovery rate was controlled at the level of 10% by applying the Benjamini-Hochberg method. *p ≤ 0.05, **p ≤ 0.01, ***p ≤ 0.001 ****p ≤ 0.0001 ns = non-significant. Data shown in A–I represent the geometric mean ± geometric SD.

The levels of antibodies against Ptx, FHA, Prn, OMVs, LOS, BrkA and Vag8 were significantly higher in plasma of symptomatic patients compared to the controls (Fig. 2A–G). The levels of antibodies against Fim2 and Fim3 did not significantly differ from controls (Fig. 2H and I). For the recovered patients, the levels of the specific antibodies for all tested antigens were significantly lower compared to that of the symptomatic patients. The Ptx-, FHA- and Prn-specific antibody levels for the recovered patients remained significantly higher than the controls (Fig. 2A–C) whereas those of OMV-, LOS-, BrkA-, Vag8-, Fim2- and Fim3-specific antibodies did not (Fig. 2D–I).

### Avidity maturation of *B. pertussis*-specific antibodies depends on antigen specificity

**Figure 3:**
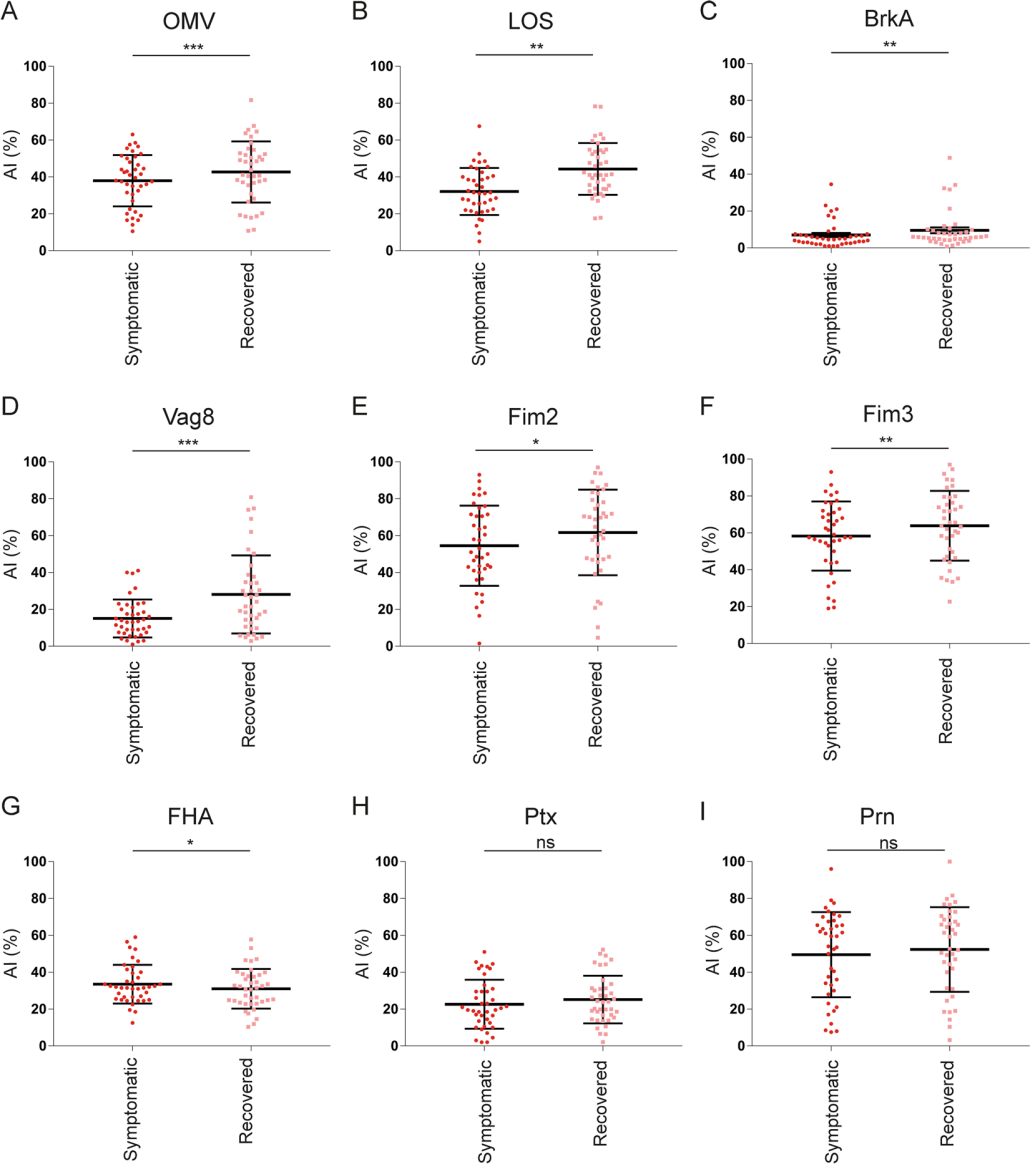
Avidity of IgG antibodies directed against nine different pertussis antigens. Avidity Index (AI) of antibodies directed against (**A**) OMVs, (**B**) LOS, (**C**) BrkA, (**D**) Vag8, (**E**) Fim2, (**F**) Fim3, (**G**) FHA, (**H**) Ptx and (**I**) Prn were determined in collected from symptomatic (circles) and recovered (squares) pertussis patients. Statistical testing: paired *t*-test. The false discovery rate was controlled at the level of 10% by applying the Benjamini-Hochberg method. *p ≤ 0.05, **p ≤ 0.01, ***p ≤ 0.001 ****p ≤ 0.0001 ns = non-significant. Data shown in A–I represent the mean ± SD.

In addition to IgG levels in plasma, we determined the avidity of antigen-specific IgG antibodies in plasma of symptomatic and recovered pertussis patients. The avidity index (AI) of the OMV-, LOS-, BrkA-, Vag8-, Fim2- and Fim3-specific antibodies were significantly higher for the recovered compared to the symptomatic patients (Fig. 3A–F). The significant difference for BrkA-IgG should be interpreted with caution as the AI is low. The AI of FHA-specific antibodies was significantly but modestly decreased for the recovered compared to the symptomatic patients (Fig. 3G). No significant difference in AI of Ptx- and Prn-specific antibodies was observed (Fig. 3H and I).

### Opsonophagocytosis wanes within years after infection but remains higher compared to controls

**Figure 4:**
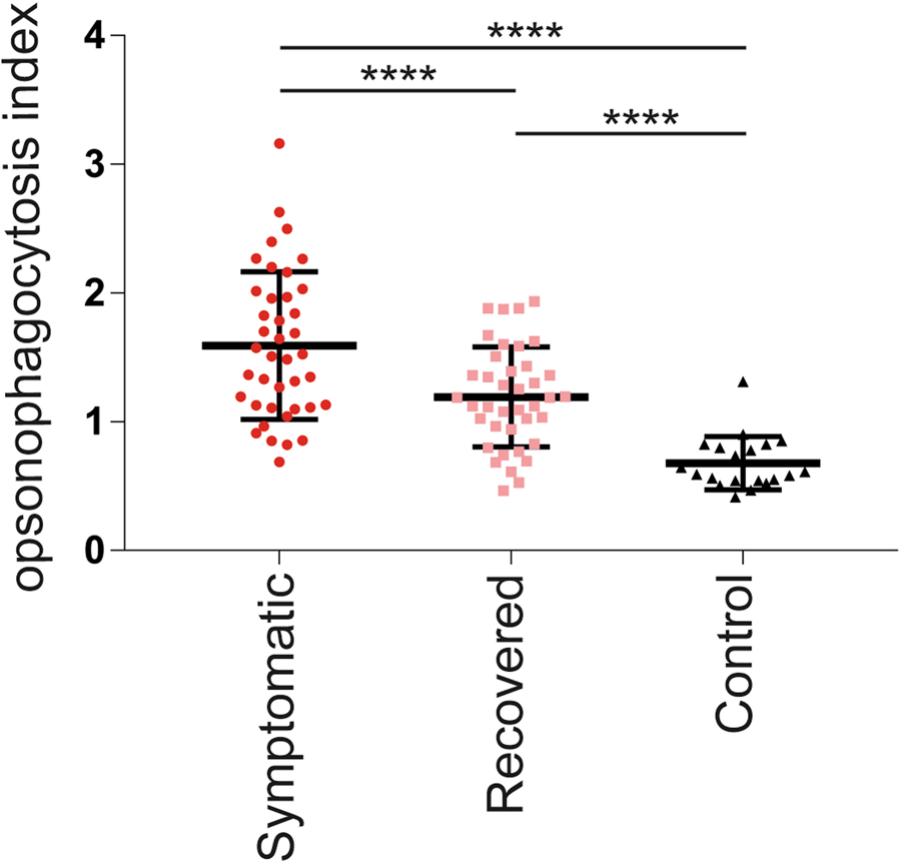
Opsonophagocytosis indices wane years after pertussis infection. Antibody-mediated opsonophagocytosis indices were determined using paired plasma samples collected from symptomatic (circles) and recovered (squares) pertussis patients. Plasma samples from controls (triangles) were also included. Statistical testing: one-way analysis of variance (ANOVA) followed by post-hoc tests (Welch Two Sample t-test or paired t-test). The false discovery rate was controlled at the level of 10% by applying the Benjamini-Hochberg method. ****p ≤ 0.0001. Data shown represent the mean ± SD.

Since antibody-mediated opsonophagocytosis is important for the clearance of *B*. *pertussis* during infection^14^, we analysed the functionality of the *B*. *pertussis*- specific antibodies using the OPA. Opsonophagocytosis by primary neutrophils was assessed using the clinical *B*. *pertussis* isolate B1917^17^, which was modified to express green fluorescent protein (GFP). We observed significantly lower opsonophagocytosis indices in plasma samples from recovered compared to symptomatic patients. Notably, the opsonophagocytosis indices from the recovered patients remained significantly higher than those of the controls (Fig. 4).

### Lower IgG levels, opsonophagocytosis indices and higher avidities of *B. pertussis*-antibodies are generally observed for recovered compared to symptomatic patients

**Figure 5:**
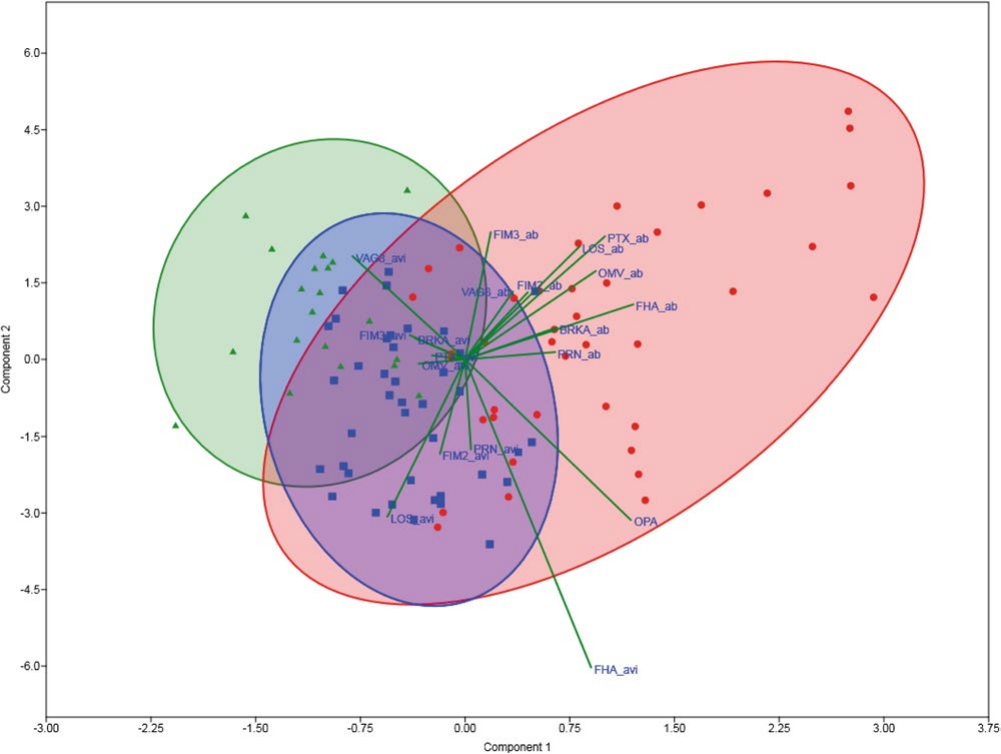
PCA of all obtained data. PCA was performed based on the antibody levels, AI and opsonophagocytosis indices for all plasma samples used in this study. The axes of differentiation of the antibody levels and AI are shown by the lines. Plasma samples collected from symptomatic patients are depicted as red dots, from recovered patients as blue squares and from controls as green triangles. The red, blue and green ellipses indicate clustering of the symptomatic, recovered and control samples, respectively. Lines indicate the axes of differentiation of the different parameters measured in this study.

In order to analyse all the obtained data, a PCA was performed based on the IgG levels and avidity as well as the opsonophagocytosis indices. As shown in Fig. 5, the three groups of the cohort form clusters, with the symptomatic patients positioned furthest away from the controls and the recovered patients being positioned in between. The lines depict the inter-correlations of the various parameters measured in all samples. Firstly, the lines indicate that the axes of differentiation of the majority of the antibody levels had similar alignments and were mainly directed towards the higher scores of the two principal components, mostly populated by the symptomatic group. Secondly, the axes of differentiation of the avidities pointed mainly in the direction of the recovered group. This analysis reflects the findings represented in Figs 2 and 3 which show lower antibody levels and higher AI in the recovered versus symptomatic patients for most of the studied antigens. Likewise, the PCA reflects the findings presented in Fig. 4 where the axes of differentiation of the opsonophagocytosis indices pointed in the direction of the symptomatic group.

### Random forest analysis reveals predictive value of FHA and Ptx antibodies for opsonophagocytosis indices

**Figure 6:**
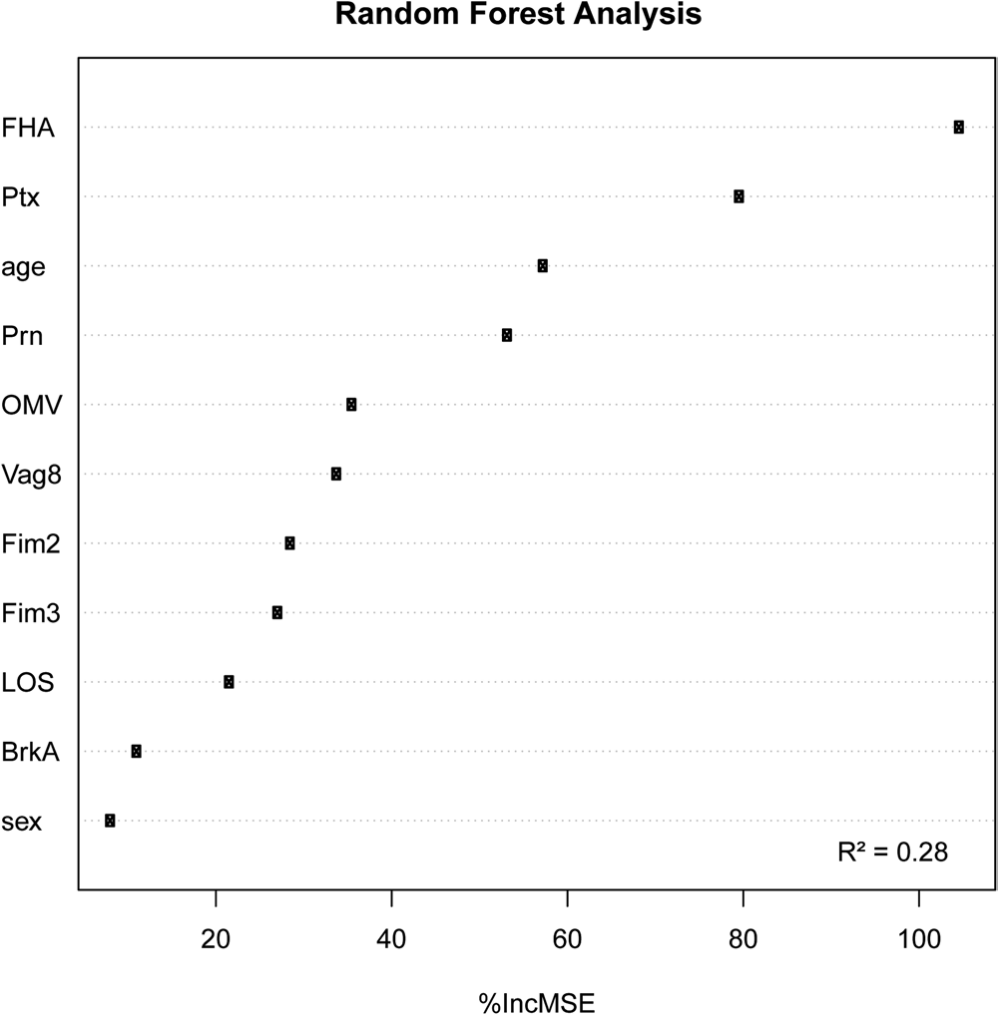
Random Forest analysis integrating all available data. Random Forest analysis was performed using a model that includes the log transformed values of the effective-IgG levels directed against the nine different antigens, as well as the age and sex of the participants. R^2^ represents the fraction of the total opsonophagocytosis indices variance which can be explained by variations in the effective-IgG levels, where a value of 1 would mean that the variance in the measured opsonophagocytosis indices is solely explained by the considered predictive variables. The percentage of increase in the mean square error (%IncMSE) after randomly permuting the values of the considered predictive variables gives an indication of how influential each considered variable is on predicting OPA values to the extent of the OPA variance which can be explained by the model (R^2^).

A random forest (RF) analysis was performed to determine which of our measured variables, namely effective-IgG levels as well as age and sex, was most predictive for the observed opsonophagocytosis and may serve as potential CoPs. The analysis showed that 28% of the variance of the opsonophagocytosis indices is explained by our model. Results indicate that FHA- followed by Ptx-specific antibodies are most predictive for the accounted variances in the opsonophagocytosis indices (Fig. 6). Multiple linear regression analysis with the same variables considered in the RF analysis revealed significant *p*-values for FHA and Ptx effective-IgGs supporting their potential predictive role (Table 1).

**Table 1:** Multi-linear regression analysis with the same parameters considered in the random forest analysis using the effective-IgG levels (IgG levels and avidity).

(log10 (IgG x Avidity)	Estimate	Std. Error	*p-*value
Prn	0.002	0.064	0.977
FHA	0.350	0.110	0.002
Fim2	−0.183	0.079	0.023
Fim3	0.172	0.085	0.045
Ptx	0.225	0.092	0.017
LOS	−0.055	0.168	0.743
Vag8	0.073	0.125	0.562
OMV	0.062	0.196	0.753
BrkA	0.117	0.088	0.187
Age	0.003	0.002	0.247
Sex	0.009	0.011	0.433

## Discussion

In the attempt to identify serological CoPs against pertussis, our study pioneers by integrating the dynamics of different antibody properties. These include their specificity to nine different *B*. *pertussis* antigens, their avidity and functional capacity to opsonise live *B*. *pertussis*. These assays were performed using paired plasma samples from symptomatic and recovered pertussis patients, who are expected to have protective immunity conferred by natural infection. As previously shown, the levels of antibodies to Ptx, FHA, Prn, Fim2, Fim3 and OMVs waned years after infection^13^. Antibodies against the other studied antigens, BrkA, Vag8 and LOS, also decreased when patients had recovered from clinical disease. BrkA, Vag8 and LOS were included in this study because they are outer membrane antigens and could be anchors for antibody-mediated phagocytosis.

Besides the waning of *B*. *pertussis-*specific antibody levels, we also show avidity maturation for the majority of specific antibodies years after *B*. *pertussis* infection with the exception of Prn, FHA and Ptx. High avidity antibodies are classically secreted by specific B cells that are positively selected during repeated exposures in germinal centre reactions^18^. In contrast to our findings, avidity maturation of Ptx antibodies was previously shown in paired samples of pertussis patients^19^. This difference may be due to the time between collections of the paired samples, which in our case was on average three years instead of four weeks after diagnosis as performed in the before mentioned study. Avidity of antibodies has also been measured following ACV booster vaccination. The authors showed that Ptx and Prn antibody avidity increased one year post-vaccination^20^. This emphasizes that in the search for CoPs against pertussis it is important to consider the antibody kinetics, not only of the levels but also of the avidity, which may differ following infection or vaccination^21^.

During an infection with *B*. *pertussis*, neutrophils infiltrate the lung where they are important for the clearance of this respiratory pathogen^14^. Here, we show that three years after infection opsonophagocytosis indices decrease but remain significantly higher compared to the controls. This is the first reported *B*. *pertussis*-specific opsonophagocytosis assay using the recently isolated *B*. *pertussis* clinical strain B1917 instead of the laboratory strain Tohama I, which has been used in the past. B1917 has the advantage of being a representative strain for the strains that were circulating at the time of our clinical study^17^. The OPA here described can discriminate between controls, symptomatic and recovered (ex) pertussis patients. Based on these findings, we propose antibody-mediated opsonophagocytosis of *B*. pertussis as a possible CoP against pertussis. Assays which measure the capacity of antibodies to opsonize pathogens for phagocytosis are currently used in the field of pneumococcal vaccination^11^. Opsonophagocytosis has also been shown to correlate with protection against malaria^22^.

Analysis of our data with the RF model using effective-IgGs levels of *B*. *pertussis*-specific antibodies uncovered FHA- followed by Ptx-antibodies as being the two variables that best predict the opsonophagocytosis indices. These findings corroborate earlier studies in which the authors also found a correlation between FHA- and to a lesser extend Ptx-specific antibodies and opsonophagocytosis^23^. In contrast to our findings, others have shown either that high anti-FHA levels inhibit opsonophagocytosis^24^ or that anti-Prn is important for opsonophagocytosis^25^. These differences may be explained by the various read-outs (bacterial uptake by flow cytometry, respiratory burst or microscopy) used to determine opsonophagocytosis, as well as the timing of serum or plasma sample collection. Effective-IgG levels to FHA, a vaccine antigen and major adhesion of *B*. *pertussis*^26^, best predicted the variances of the opsonophagocytosis indices. FHA-antibodies are expected to bind to the bacterial membrane and mediate bacterial uptake by engaging Fc-γ receptors on neutrophils^27^. It remains unknown whether FHA-specific antibodies generated upon infection and vaccination are similar in functionality. Antibodies against the additionally included outer membrane-localised antigens LOS^28^, Vag8 and BrkA^29,30^, important immune modulators mediating resistance against antimicrobial peptides (LOS) and complement (LOS, Vag8 and BrkA), were not predictive for the opsonophagocytosis indices according to the RF analysis. It is possible that these antibodies are of an IgG subclass that does not mediate opsonophagocytosis or that the antigen is masked on the bacterial membrane. However, this does not exclude other functional roles for human antibodies against these additional surface antigens. Monoclonal antibodies directed against LOS were found to be important in preventing *B*. *pertussis* infection in a mouse model^31^ and BrkA-specific antibodies have been implicated in bactericidal killing of *B*. *pertussis*^32^. Further studies should address these matters. The second best predictor for opsonophagocytosis was the presence of antibodies directed to Ptx. Because this toxin is generally secreted by *B*. *pertussis* and induces high levels of antibodies following infection^16,33,34^, we speculate that the association found in the RF model is not mechanistically related. Interestingly, FHA- and Ptx-specific antibodies did not show an increased avidity in the recovered pertussis patients suggesting that perhaps high antibody avidity for these antigens is not necessarily important for opsonophagocytosis of *B*. *pertussis*. Whether the FHA, and possibly Ptx, antibodies are mechanistically related to neutrophil opsonophagocytosis will have to be verified by either depleting or and/or isolating these antibodies from plasma, as well as by performing the OPA with *B*. *pertussis* that do not express FHA or Ptx. To determine whether these antibodies indeed also protect from colonisation with *B*. *pertussis*, an *in vitro* model using differentiated human airway epithelial cells^35,36^ may be used for validation. Moreover, eventually the novel controlled human infection pertussis model^37^ could be valuable to determine protection from colonization by specific antibodies.

Although FHA- and Ptx- specific antibodies were the best predictors for opsonophagocytosis in our model, their presence only explained 28% of the opsonophagocytosis indices variances. Adenylate cyclase toxin (ACT) produced by *B*. *pertussis* reduces phagocytosis and subsequent bacterial killing by neutrophils^14,38^. Previously, antibodies directed against this toxin have been shown to promote phagocytosis of *B*. *pertussis* via toxin-neutralization^39^. ACT is not only secreted but also remains associated to the bacterial membrane by interacting with FHA^40,41^. ACT-specific antibodies could hence also opsonize *B*. *pertussis*. Although ACT was not included in the antigen panel of this study, it is likely that part of the unexplained variances can be attributed to ACT-specific antibodies. Also, other specificities cannot be excluded, as the naturally acquired or vaccine-induced *B*. *pertussis* serum reactomes are very diverse and broad^42^. Future studies should explore whether ACT-specific or other *B*. *pertussis-*specific antibodies further predict opsonophagocytosis.

This work represents a first step towards identifying CoPs by the combined analysis of different parameters of *B*. *pertussis*-specific antibodies from (ex) pertussis patients. These findings should be interpreted with caution as the longitudinal pertussis cohort used is small and includes individuals of different ages with different vaccination status and most likely previous infection backgrounds. Due to the low sample size we were unable to analyse our data by grouping the individuals according to age. Furthermore, our cohort does not include young infants whom are the main victims of severe pertussis. The induction of *B*. *pertussis-*specific antibodies and their functional properties may differ between infants and the older individuals in our cohort, possibly due to the presence of maternal antibodies^43^, which should be taken into account when developing novel childhood pertussis vaccines.

We furthermore suggest exploring antibody characteristics such as subclass and fucosylation status^44^ which can influence opsonizing capacity or other antibody effector functions. Additionally, protective mechanisms including neutrophil respiratory burst^45^ and subsequent killing, as well as complement-mediated killing of *B*. *pertussis*^46–48^ could be studied. Recently, levels of specific memory B cells, that can give rise to a fast increase in antibody levels upon an infection with *B*. *pertussis*, were implied in protection^20,49,50^. Moreover, it is clear that not only antibodies or B cells are involved in protection against pertussis, as T helper 1(Th1) and Th17 cells^49,51^ have also been implicated as very important players in the protective immune response against *B*. *pertussis*. Taken together, the pertussis field should broaden the way identification of CoPs is approached and be open to multifactorial CoPs against pertussis, which could be a combination of antibody levels, antibody functionality as well as *B*. *pertussis* specific T and B cell responses. With respect to antibodies, integration of different specific antibody parameters may bring the field closer to the identification of the required CoPs against pertussis.

## Materials and Methods

### Ethics

Participants donating blood for plasma isolation were Dutch symptomatic (ex-) pertussis patients selected from a cross-sectional observational study previously described (Specifieke Kinkhoest Immuniteit; SKI)^13^. The study was approved by the accredited Medical Research Ethics Committee (MREC) STEG followed by management of the METC UMC Utrecht (Dutch Central Committee on Research Involving Human Subjects (CCMO) nr: NL16334.040.07). Collection of all samples used was conducted according to the principles of the Declaration of Helsinki and all participants, parents/guardians of minor participants and blood donors for primary cell isolation provided written informed consent. Samples were treated anonymously.

### Study population

Forty individuals (age 7–73 years, median 49.4 years, at inclusion; male/female ratio: 0.35/0.65) who presented clinical pertussis symptoms and were physician-diagnosed with pertussis, either by PCR or serology, were selected from the SKI cohort, a Dutch natural infection cohort established between 2008 and 2012^13^. Selection was based on whether two longitudinal samples were available namely, early after diagnosis (1.7 to 9.0 weeks, median 3.15 weeks, i.e. symptomatic) when the patients still presented pertussis symptoms and years later (2.7 to 4.7 years, median 3.2 years, i.e. recovered) when the patients had recovered from clinical disease. An additional 20 household contacts from the SKI cohort (age 7.9–48.2 years, median 21.4 years; male/female ratio 0.5/0.5, i.e. controls) who were seronegative (negative anti-Ptx titres) for pertussis and vaccinated according to the Dutch national immunisation program were also selected (Fig. 1).

### Plasma collection and neutrophil isolation

Blood samples in the SKI study were collected using vacutainer CPT cell preparation tubes (BD Biosciences, San Jose, CA, USA). Plasma was isolated using standard procedures and stored at −80 °C. Plasma was heat inactivated (HI) for 20 minutes at 56 °C. Human neutrophils were isolated from donor blood as described previously^52^. Briefly, heparinized venous blood obtained from healthy donors in vacutainer collection tubes (BD Biosciences) was twice diluted in PBS (Gibco) and layered onto a gradient of Ficoll-Paque Premium (GE Healthcare Life Sciences) and Histopaque-1119 (Sigma-Aldrich). After centrifugation for 20 minutes at 400 g, neutrophils were collected from the Histopaque layer and washed with RPMI-1640 (Gibco), supplemented with 0.05% human serum albumin (HSA, Sanquin). Neutrophils were used in the opsonophagocytosis assay shortly after isolation.

### Bacteria

*B*. *pertussis* B1917^17^ was transformed with plasmid pCW505 resulting in cytoplasmic expression of GFP (B1917-GFP)^25^. Bacteria were grown on Bordet-Gengou agar plates containing 15% sheep blood (BD Biosciences) supplemented with 30 µg/ml gentamycin (Merck, Darmstadt, Germany) at 35 °C, 5% CO_2_ for four days. Subsequently, bacteria were recultured in Thalen-IJssel medium^53^ overnight (start OD 0.05, 35 °C, 130 rpm) until mid-log phase (OD 0.7) was reached before use.

### Pertussis antigens

Ptx and FHA were obtained from Kaketsuken (Obuko, Japan). Fim2 was a kind gift from Dr. A Gorringe, Fim3 and Prn were purified in-house. LOS was extracted from strain B1917 by means of hot phenol-water as previously described^54^. OMVs of B1917, containing various outer membrane antigens, were generated as previously described^55^ with some modifications and recombinant passenger domains of Vag8 and BrkA were expressed in *Escherichia*. *coli* as described elsewhere^47^.

### Multiplex immunoassay

Levels of total IgG directed against *B*. *pertussis* antigens Ptx, FHA, Prn, Fim2, Fim3, Vag8, BrkA, LOS and OMV were measured in HI-plasma samples by an in-house multiplex immunoassay (MIA) described earlier^13^. The International WHO pertussis standard (NIBSC 06/140) served as reference for IgG levels against Ptx, FHA and Prn (International units, IU/ml). Human Normal Immunoglobulin solution for infusion (KIOVIG, Baxalta, Belgium) was used as an in-house reference to measure levels of IgG against the remaining six antigens, expressed as arbitrary units per ml (AU/ml) based on observed relative magnitudes of fluorescence intensity where KIOVIG was set at 500 AU/ml. For detection, goat-anti-human IgG-PE (Jackson ImmunoResearch, USA) was used. Analysis was performed with a Bio-Plex 200 using Bio-Plex Manager software version 5.5 (Bio-Rad Laboratories, West Grove, PA, USA). The various antigen coupled bead regions were not found to interfere with one another.

### Avidity assay

The avidity of pertussis-specific IgG antibodies was measured in the MIA using HI-plasma as described previously^56^ with modifications. Briefly, plasma samples were allowed to bind for 45 minutes with the respective pertussis antigen coupled to fluorescently labelled microspheres. After washing, samples were incubated for 15 minutes in PBS either in the absence or presence of 9 M urea, to elute lower-avidity antibodies. Subsequently, samples were washed, goat-anti-human IgG-PE (Jackson ImmunoResearch, USA) was used as detection antibody and analysis was performed with a Bio-Plex 200 using Bio-Plex Manager software version 5.5 (Bio-Rad) The avidity index (AI) was expressed as a percentage of the remaining IgG levels in the presence of urea relative to the IgG levels measured in PBS only.

### Opsonophagocytosis assay

Opsonophagocytosis of *B*. *pertussis* B1917 by human neutrophils was evaluated using a method described in literature with minor modifications^27,57^. Briefly, B1917-GFP (multiplicity of infection is 70) were opsonized with 5% HI-plasma in RPMI supplemented with 0.3% human serum albumin (HSA, Sanquin) for 20 minutes at 37 °C. Subsequently, freshly isolated neutrophils (7.5 × 10^4^ cells) were added in a total volume of 50 µl and incubated for 25 minutes at 4 °C. After a wash step with cold RPMI-HSA, neutrophils were resuspended in RPMI-HSA and incubated for 30 minutes at 37 °C. The incubation times used were selected based on OPA pilot experiments. Cells were fixed with 1.5% paraformaldehyde and visualized using the FACSCanto II (BD Bioscience). The assay was performed in duplicate on three different days. To correct for plate and day differences, a standalone-control plasma sample was taken along on each plate for each day and a plate factor was calculated. The arbitrary opsonophagocytosis indices were calculated by dividing the mean fluorescent intensity (MFI) obtained in the presence of plasma by the MFI obtained upon incubating cells with bacteria without plasma, corrected for the plate factor.

### Data analysis

Differences in antibody levels and opsonophagocytosis indices between the groups were tested with one-way analysis of variance (ANOVA) followed by post-hoc tests (Welch Two Sample *t-*test or paired *t*-test). For differences in the AI a paired *t*-test was performed. P values ≤ 0.05 were considered statistically significant. The false discovery rate was controlled at the level of 10% by applying the Benjamini-Hochberg method to the results of all tests (Supplementary Table 2).

Principal component analysis (PCA) was used to explore dis(similarities) among the antibody levels, AI and opsonophagocytosis indices of the three groups.

To determine which of the measured antibodies were most predictive for the observed opsonophagocytosis, a random forest (RF) analysis was performed^58^. The model considered in this analysis uses for each antigen the effective-IgG level, which we define as the logarithm of the product of IgG levels and AI (IgG*AI). A multiple linear regression analysis using the same effective-IgG levels was also performed. Descriptive statistics of the data can be found in Supplementary Table 1.

## Electronic supplementary material

Supplementary information
