# Supplementary material for: Detection of opsonizing antibodies directed against a recently circulating Bordetella pertussis strain in paired plasma samples from symptomatic and recovered pertussis patients
Source: Sci Rep. 2018 Aug 13;8:12039. doi: 10.1038/s41598-018-30558-8 (PMC6089961; doi:10.1038/s41598-018-30558-8)
Supplement: Supplementary file 1 — Supplementary information [file 41598_2018_30558_MOESM1_ESM.pdf]

Detection of opsonizing antibodies directed against a recently circulating *Bordetella pertussis* strain in paired plasma samples from symptomatic and recovered pertussis patients.

Elise S. Hovingh[1,2], Betsy Kuipers[1], Axel A. Bonačić Marinović[1], Hendrik Jan Hamstra[1], Danielle Hijdra[1], Lapo Mughini Gras[1,3], Inonge van Twillert[1], Ilse Jongerius[1,2,$], Cecile A.C.M van Els[1*] and Elena Pinelli[1*,#]

[1]Centre for Infectious Disease Control, National Institute for Public Health and the Environment (RIVM), Bilthoven, The Netherlands. [2]Department of Medical Microbiology, University Medical Centre Utrecht, Utrecht, The Netherlands. [3]Department of Infectious Diseases and Immunology, Utrecht University, Utrecht, The Netherlands.

[*]These senior authors contributed equally to this manuscript.

$ Currently based at Department of Immunopathology, Sanquin Research and Landsteiner Laboratory of the Academic Medical Centre, University of Amsterdam, Amsterdam, The Netherlands

Supplementary Table 1: Description of data presented in this study.

	Mean	Standard Deviation	Minimum	Maximum
**All samples** (n=100)				
OPA	1,24	0,56	0,42	3,16
Anti-Ptx	116,22	185,47	1,20	1257,45
Anti-FHA	201,89	205,49	17,65	1214,05
Anti-Prn	91,50	184,54	0,35	1264,05
Anti-Fim2	31,34	70,59	0,25	368,70
Anti-Fim3	11,27	29,67	0,25	270,90
Anti-OMVs	38,51	47,58	1,65	250,20
Anti-LOS	70,40	106,21	2,00	528,70
Anti-Vag8	47,71	117,73	1,70	982,05
Anti-BrkA	89,64	167,70	0,45	1121,20
AI-Ptx	0,24	0,12	0,02	0,52
AI-FHA	0,29	0,23	0,07	0,59
AI-Prn	0,50	0,23	0,03	1,13
AI-Fim2	0,59	0,19	0,02	1,03
AI-Fim3	0,63	0,16	0,19	1,01
AI-OMVs	0,41	0,16	0,11	0,82
AI-LOS	0,39	0,15	0,05	0,91
AI-Vag8	0,26	0,25	0,01	1,79
AI-BrkA	0,09	0,09	0,01	0,49
**Symptomatic** (n=40)				
OPA	1,59	0,57	0,69	3,16
Anti-Ptx	245,185	23,9034	31,15	1257,45
Anti-FHA	361,1975	236,8367	52,8	1214,05
Anti-Prn	165,2662	255,435	1,15	1264,35
Anti-Fim2	53,86	101,0237	0,3	368,7
Anti-Fim3	17,0962	43,69909	0,3	270,9
Anti-OMVs	68,585	61,1552	9,35	250,2
Anti-LOS	131,7713	143,465	3,95	528,7
Anti-Vag8	77,68625	174,0224	1,8	982,05
Anti-BrkA	160,585	231,405	0,45	1121,2
AI-Ptx	0,22633	0,1133	0,0175	-0,5106
AI-FHA	0,33549	0,1047	0,1281	0,5884
AI-Prn	0,49643	0,2335	0,0717	1,0164
AI-Fim2	0,54758	0,2212	0,01557	0,9998
AI-Fim3	0,58314	0,1891	0,1925	0,9669
AI-OMVs	0,3792	0,1394	0,105612	0,6313
AI-LOS	0,32145	0,1277	0,0471	0,6769
AI-Vag8	0,15128	0,1023	0,0111	0,4097
AI-BrkA	0,0696	0,071	0,0066	0,3417
**Recovered** (n=40)				
OPA	1,18	0,40	0,47	1,94
Anti-Ptx	38,3475	54,439	4,1	334,45
Anti-FHA	121,75	71,23	28,6	330
Anti-Prn	58,085	105,1909	0,55	560,9
Anti-Fim2	17,0325	36,0705	0,25	162,7
Anti-Fim3	5,14625	7,3895	0,25	28,2

Anti-OMVs	21,41375	19,6239	2,6	85,45
Anti-LOS	32,24875	34,58	2,95	125,45
Anti-Vag8	27,05625	34,307	1,7	161,3
Anti-BrkA	53,645	92,057	0,5	425,1
AI-Ptx	0,25248	0,1294	0,0224	0,521
AI-FHA	0,31083	0,1074	0,1053	0,5757
AI-Prn	0,52677	0,237	0,0341	1,1317
AI-Fim2	0,62404	0,2404	0,0482	1,0309
AI-Fim3	0,640905	0,1936	0,2268	1,0144
AI-OMVs	0,42651	0,166	0,1057	0,8152
AI-LOS	0,4429	0,1401	0,1773	0,7821
AI-Vag8	0,28077	0,2123	0,0279	0,806
AI-BrkA	0,951285	0,1015	0,0073	0,4896
**Controls** (n=20)				
OPA	0,68	0,21	0,42	1,31
Anti-Ptx	14,03	12,2024	1,2	42,45
Anti-FHA	43,5725	33,6289	17,65	139,35
Anti-Prn	10,795	11,9578	0,35	43,8
Anti-Fim2	14,92	23,7119	0,45	93,95
Anti-Fim3	11,86	20,1178	0,6	74,2
Anti-OMVs	12,557	9,897	1,65	31,75
Anti-LOS	23,957	28,6594	2	119,95
Anti-Vag8	29,04	66,9428	2,85	287,65
Anti-BrkA	19,7275	27,3995	0,95	99,85
AI-Ptx	0,2668	0,1295	0,0819	0,5248
AI-FHA	0,18	0,0828	0,0693	0,3312
AI-Prn	0,4736	0,2096	0,2086	0,9181
AI-Fim2	0,5902	0,2088	0,1789	0,9712
AI-Fim3	0,694	0,1774	0,2794	0,9156
AI-OMVs	0,4471	0,1714	0,2173	0,8188
AI-LOS	0,4215	0,1829	0,1743	0,9126
AI-Vag8	0,4294	0,4022	0,0418	1,7859
AI-BrkA	0,1139	0,1057	0,0133	0,3726

Supplementary Table 2: Statistics presented in this study.

k	Group 1	Group 2	Outcome variable	p.value	transformed.p.value (FDR)		number of tests	no. false positives
1	Symptomatic	Recovered	IgG PTX	0.00000	0.00000	****	39	0.000
2	Symptomatic	Control	IgG FHA	0.00000	0.00000	****		0.000
3	Symptomatic	Recovered	IgG FHA	0.00000	0.00000	****		0.000
4	Symptomatic	Recovered	IgG OMV	0.00000	0.00000	****		0.000
5	Symptomatic	Recovered	IgG LOS	0.00000	0.00000	****		0.000
6	Symptomatic	Recovered	IgG PRN	0.00000	0.00000	****		0.000
7	Symptomatic	Recovered	IgG FIM3	0.00000	0.00000	****		0.000
8	Symptomatic	Control	IgG PTX	0.00000	0.00000	****		0.000
9	Symptomatic	Recovered	IgG FIM2	0.00000	0.00001	****		0.000
10	Symptomatic	Recovered	OPA	0.00001	0.00004	****		0.000
11	Symptomatic	Control	IgG OMV	0.00003	0.00010	****		0.001
12	Control	Recovered	IgG FHA	0.00006	0.00020	****		0.002
13	Symptomatic	Control	OPA	0.00010	0.00030	****		0.004
14	Control	Recovered	OPA	0.00010	0.00028	****		0.004
15	Symptomatic	Control	IgG BRKA	0.00035	0.00090	***		0.014
16	Symptomatic	Control	IgG PRN	0.00039	0.00096	***		0.015
17	Symptomatic	Recovered	AI VAG8	0.00088	0.00203	***		0.034
18	Symptomatic	Recovered	IgG VAG8	0.00127	0.00274	**		0.049
19	Symptomatic	Recovered	AI LOS	0.00144	0.00296	**		0.056
20	Symptomatic	Recovered	AI BRKA	0.00163	0.00318	**		0.064
21	Control	Recovered	IgG PTX	0.00193	0.00359	**		0.075
22	Control	Recovered	IgG PRN	0.00257	0.00456	**		0.100
23	Symptomatic	Recovered	AI FIM3	0.00263	0.00445	**		0.102
24	Symptomatic	Recovered	IgG BRKA	0.00279	0.00453	**		0.109
25	Symptomatic	Control	IgG LOS	0.00618	0.00963	**		0.241
26	Symptomatic	Control	IgG VAG8	0.00642	0.00963	**		0.250
27	Symptomatic	Recovered	AI OMV	0.01126	0.01626	*		0.439
28	Symptomatic	Recovered	AI FIM2	0.01484	0.02067	*		0.579
29	Symptomatic	Recovered	AI FHA	0.02013	0.02707	*		0.785
30	Control	Recovered	IgG OMV	0.04673	0.06075			1.822
31	Symptomatic	Recovered	AI PTX	0.07351	0.09248			2.867
32	Control	Recovered	IgG BRKA	0.08220	0.10018			3.206
33	Symptomatic	Control	IgG FIM2	0.10110	0.11948			3.943
34	Symptomatic	Recovered	AI PRN	0.12920	0.14820			5.039
35	Symptomatic	Control	IgG FIM3	0.27900	0.31089			10.881
36	Control	Recovered	IgG FIM3	0.30820	0.33388			12.020
37	Control	Recovered	IgG VAG8	0.31690	0.33403			12.359
38	Control	Recovered	IgG LOS	0.40950	0.42028			15.971
39	Control	Recovered	IgG FIM2	0.79090	0.79090			30.845
